# Radiotherapy for inferior vena cava tumor thrombus in patients with hepatocellular carcinoma

**DOI:** 10.1186/s12885-019-5654-9

**Published:** 2019-06-10

**Authors:** Tzu-Hui Pao, Wei-Ting Hsueh, Wei-Lun Chang, Nai-Jung Chiang, Yih-Jyh Lin, Yi-Sheng Liu, Forn-Chia Lin

**Affiliations:** 1 Department of Radiation Oncology, National Cheng Kung University Hospital, College of Medicine, National Cheng Kung University, No.138, Sheng Li Road, Tainan, 70456 Taiwan; 2 Department of Internal Medicine, National Cheng Kung University Hospital, College of Medicine, National Cheng Kung University, Tainan, Taiwan; 3 Institute of Clinical Medicine, College of Medicine, National Cheng Kung University, Tainan, Taiwan; 4 National Institute of Cancer Research, National Health Research Institutes, Tainan, Taiwan; 5 Division of Hematology/Oncology, Department of Internal Medicine, National Cheng Kung University Hospital, Tainan, Taiwan; 6 Division of Transplant surgery, Department of Surgery, National Cheng Kung University Hospital, College of Medicine, National Cheng Kung University, Tainan, Taiwan; 7 Department of Diagnostic Radiology, National Cheng Kung University Hospital, College of Medicine, National Cheng Kung University, Tainan, Taiwan

**Keywords:** Hepatocellular carcinoma, Inferior vena cava thrombus, Radiation therapy

## Abstract

**Background:**

Hepatocellular carcinoma (HCC) with inferior vena cava (IVC) involvement is a rare disease with poor prognosis. This study aimed to evaluate the outcome of HCC patients receiving radiotherapy (RT) to IVC tumor thrombus.

**Methods:**

A total of 42 consecutive HCC patients treated with RT to IVC tumor thrombus between September 2007 and October 2018 were enrolled. Overall survival (OS), the response of IVC thrombus, prognostic factors and failure pattern were assessed.

**Results:**

The median follow-up time was 4.4 months. The median RT equivalent dose in 2-Gy fractions was 48.75 Gy (range, 3.25–67.10). The objective response rate of IVC thrombus was 47.6% (95% confidence interval [CI], 33.3–64.3%). The OS rate at 1 year was 30.0%, with a median OS of 6.6 months (95% CI, 3.7–9.5) from the start of RT. On multivariate analysis, Child-Pugh class, lymph node metastasis, lung metastasis and objective response of IVC thrombus were independent predictors for OS. Lung was the most common site of first progression in 14 (33.3%) patients. For 32 patients without lung metastasis before RT, use of systemic treatment concurrent with and/or after RT was associated with a significantly longer lung metastasis-free survival (5.9 vs. 1.5 months, *p* = 0.0033).

**Conclusions:**

RT is effective for IVC tumor thrombus of HCC with acceptable adverse effects. RT might be a treatment option incorporated into combination therapy for HCC involving IVC.

## Introduction

Hepatocellular carcinoma (HCC) is the most common type of liver cancer and the third ranked cause of global cancer mortality [1]. Vascular invasion is a prognostic factor for poor overall survival (OS) in patients with HCC [2, 3]. Compared to portal and hepatic veins, inferior vena cava (IVC) was less frequently involved by HCC. IVC tumor thrombus may flow into heart and lung, leading to pulmonary embolism and lung metastasis. Patients with HCC involving IVC had an increased risk of sudden death and dismal treatment outcome [4, 5].

HCC involving IVC is difficult to treat and a standard therapy has not been established. Surgery, transarterial chemoembolization (TACE) and systemic treatment were adopted in the management of these cases. The use of radiotherapy (RT) remains controversial. Some retrospective studies have suggested that RT is a feasible and safe option to palliate HCC with IVC invasion with pooled 1-year OS rate of 53.6%, response rate of 59.2% and possible severe complication rate of 1.2% [6]. However, the data regarding lung metastasis and pulmonary embolism after RT for these patients was limited.

In the current study, we retrospectively evaluated the clinical outcomes and prognostic factors in HCC patients receiving RT to IVC tumor thrombus. In addition, the information of lung metastasis and pulmonary embolism before and after RT was reported.

## Methods

### Patients

This study enrolled 42 consecutive HCC patients receiving RT to IVC tumor thrombus at our hospital from September 2007 to October 2018. The demographic and clinical features, treatment modalities and outcomes of these patients were gathered from a review of the medical records. HCC was diagnosed on the basis of histological examination or the image criteria of the American Association for the Study of Liver Diseases guideline [7]. The IVC tumor thrombus was diagnosed by characteristic finding of computed tomography (CT) or magnetic resonance imaging (MRI). All patients had pretreatment assessment consisted of a history and physical examination, hematology, biochemistry, Hepatitis B/C panel and chest radiographs.

### Radiotherapy

Radiotherapy was delivered by using a linear accelerator with intensity-modulated radiotherapy (IMRT) in 35 patients and three dimensional conformal radiation therapy (3DCRT) in seven patients. All patients were immobilized by customized devices in the supine position with both arms raised above the head. The simulation CT scan was acquired at 5 mm slice thickness and transferred for treatment planning system to determine the radiation volume and dose distribution. RT was planned to target IVC and right atrium thrombus. Synchronously, hepatic or portal vein thrombus was also irradiated in 26 patients and intrahepatic tumor in seven patients according to the suggestion of our institutional multidisciplinary HCC team. The gross tumor volume (GTV) was defined as the hypodense filling defect area for venous thrombus and the hyperdense area for intrahepatic tumors. The clinical target volume (CTV) was defined as GTV plus a 0.5–1 cm margin along the vein for thrombi and in all directions for intrahepatic tumors. For cases with tumor thrombus in the right atrium, we included the whole right atrium in the CTV. The planning target volume (PTV) was determined by adding a 0.5–1 cm margin to the CTV for uncertainties in treatment delivery. A daily dose of 2–3 Gy was delivered to the PTV using 6- or 10- MV X-rays at five fractions per week. The RT dose was converted to equivalent dose in 2-Gy fractions for α/β = 10 (EQD_10/2_). Other therapies for HCC given within 4 weeks before or after RT was defined as concurrent treatments.

### Response assessments and follow-up

CT or MRI scans were performed at 1 to 3 months after the completion of RT and then every 3 to 6 months thereafter. The response of the IVC tumor thrombus was assessed according to the World Health Organization criteria [8]. The product of the two greatest perpendicular diameters of the IVC tumor thrombus was calculated and compared with the baseline value. Complete disappearance of IVC tumor thrombus was defined as complete response (CR), decrease of ≥50% in IVC thrombus size as partial response (PR), decrease of <50% in IVC tumor thrombus or increase of < 25% as stable disease (SD), and increase of ≥25% as progressive disease (PD). The objective response included CR and PR. Adverse effects were graded according to the Common Terminology Criteria for Adverse Events (CTCAE; version 3.0).

### Statistical methods

The data cutoff date was October 10, 2018. The survival curves were estimated using the Kaplan-Meier method and compared statistically using the log-rank test. OS was calculated from the start of RT to the date of death. For patients without lung metastasis before RT, lung metastasis-free survival was measured from the start of RT to the date of death or the development of lung metastasis. Univariate and multivariate Cox proportional hazards analyses were performed to check factors associated with OS and lung metastasis-free survival. A *p* value of < 0.05 was considered statistically significant. Data analysis was performed with SPSS version 22.0 software and R version 3.5.1 for Windows.

## Results

### Patient characteristics

The demographic and clinical characteristics of 42 patients at baseline are summarized in Table 1. The cohort included 29 men (69%) and 13 women (31%). The majority of patients (90.5%) had hepatitis B and/or C. Eight (19.0%) patients had lymph node (LN) metastasis and ten (23.8%) patients had lung metastasis. In addition to IVC, thrombus was also noted in right atrium in 11 (26.2%), portal vein in 16 (38.1%), and hepatic vein in 15 (35.7%) patients.

**Table 1:** Demographic and Clinical Characteristic of Patients at Baseline

Characteristic	No. of patients (%)
Age (years)	
Median	63
Range	40–92
Gender	
Male	29 (69.0)
Female	13 (31.0)
ECOG performance status	
0	4 (9.5)
1	23 (54.8)
2	14 (33.3)
3	1 (2.4)
Child-Pugh class	
A	25 (59.5)
B	15 (35.7)
C	2 (4.8)
Etiology of chronic liver disease	
HBV	24 (57.1)
HCV	11 (26.2)
HBV & HCV	3 (7.1)
Others	4 (9.5)
Thrombus site besides IVC^a^	
Right atrium	11 (26.2)
Portal vein	16 (38.1)
Hepatic vein	15 (35.7)
LN metastasis	
Absent	34 (81.0)
Present	8 (19.0)
Lung metastasis	
Absent	32 (76.2)
Present	10 (23.8)

Abbreviations: *ECOG* Eastern Cooperative Oncology Group, *HBV* hepatitis B virus, *HCV* hepatitis C virus, *IVC* inferior vena cava, *LN* lymph node

^a^Patients with multiple thrombus site besides IVC were counted for each site

### Treatment characteristics

All treatment modalities are summarized in Table 2. Before RT to IVC thrombus, ten patients were treated by surgery, 19 by TACE/TAE (transarterial embolization), and 11 by radiofrequency ablation (RFA) or percutaneous ethanol injection (PEI). Thirty-six patients completed the RT treatment while RT was terminated earlier in six patients (3 due to terminal liver disease, 2 pulmonary embolism and 1 respiratory failure). The median RT EQD_10/2_ were 48.75 Gy (range, 3.25–67.10). Within 4 weeks before or after RT, 13 patients underwent TAE/TACE or RFA/PEI for their intrahepatic tumors. Furthermore, twelve (28.6%), 24 (57.1%) and 21 (50.0%) patients received individualized systemic treatment before, during and after RT, respectively (Table 3). Sorafenib was the most commonly used systemic anticancer drug, with a median treatment duration of 3.3 months in 31 patients.

**Table 2:** Treatment Characteristics

Treatment	No. of patients (%)
Previous treatment^a^	
None	15 (35.7)
Operation	10 (23.8)
TACE/TAE	19 (45.2)
RFA/PEI	11 (26.2)
Systemic treatment	12 (28.6)
Treatment concurrent with RT^b^	
None	13 (31.0)
Operation	0 (0.0)
TACE/TAE	12 (28.6)
RFA/PEI	1 (2.4)
Systemic treatment	24 (57.1)
Treatment after RT^c^	
None	20 (47.6)
Operation	4 (9.5)
TACE/TAE	6 (14.3)
RFA/PEI	1 (2.4)
Systemic treatment	21 (50.0)
RT technique	
3DCRT	7 (16.7)
IMRT	35 (83.3)
RT dose (EQD_10/2_, Gy)^d^	
Median (range)	48.75 (3.25–67.10)
< 50	22 (52.4)
≥ 50	20 (47.6)

Abbreviations: *RT* radiotherapy, *TACE* transarterial chemoembolization, *TAE* transarterial embolization, *RFA* radiofrequency ablation, *PEI* percutaneous ethanol injection, *3DCRT* three-dimensional conformal radiotherapy, *IMRT* intensity modulation radiation therapy

^a^Treatment given beyond 4 weeks before RT began

^b^Treatment given within 4 weeks before RT began or 4 weeks after RT completed

^c^Treatment given beyond 4 weeks after RT completed

^d^Equivalent dose in 2 Gy fractions, α/β = 10

**Table 3:** Systemic Therapy

	No. of patients (%)		
Systemic therapy	Pre-R/T	During RT	Post-R/T
Total	12 (28.6)	24 (57.1)	21 (50.0)
Sorafenib	12 (28.6)	17 (40.5)	16 (38.1)
Nivolumab	1 (2.4)	2 (4.8)	2 (4.8)
Thalidomide	0 (0.0)	4 (9.5)	1 (2.4)
Tegafur/uracil	0 (0.0)	1 (2.4)	0 (0.0)
Ramucirumab	0 (0.0)	0 (0.0)	3 (7.1)
Everolimus	0 (0.0)	0 (0.0)	1 (2.4)
Pegargiminase	0 (0.0)	0 (0.0)	1 (2.4)

### IVC response and survival outcomes

**Fig. 1:**
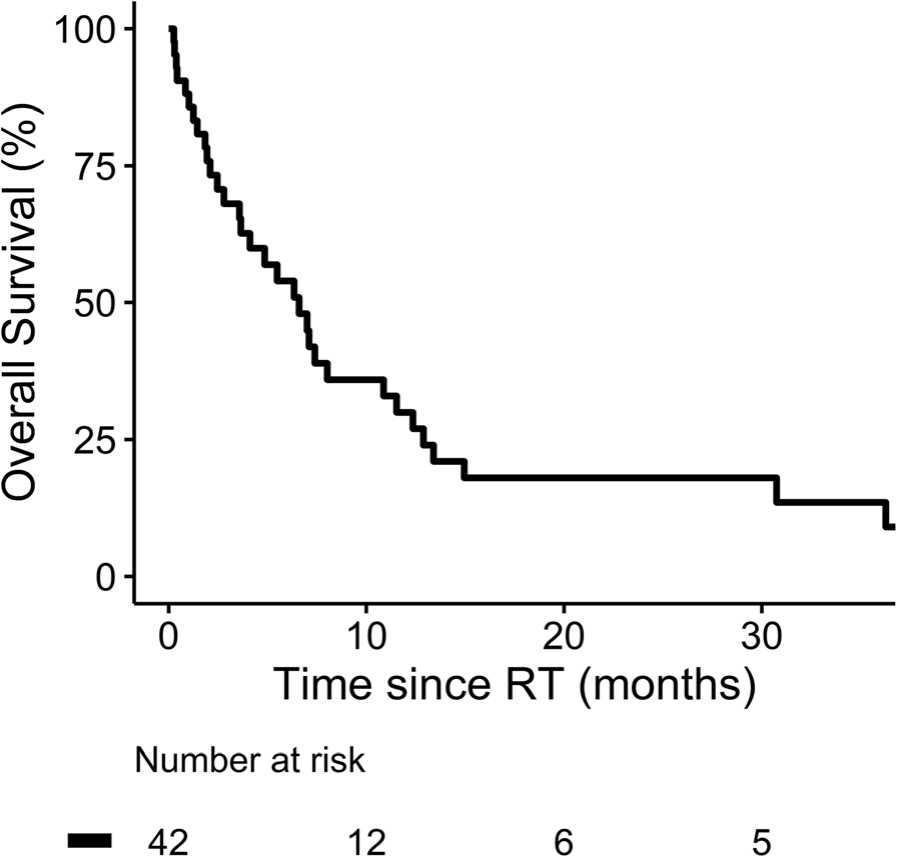
Overall survival for whole group of 42 patients

**Fig. 2:**
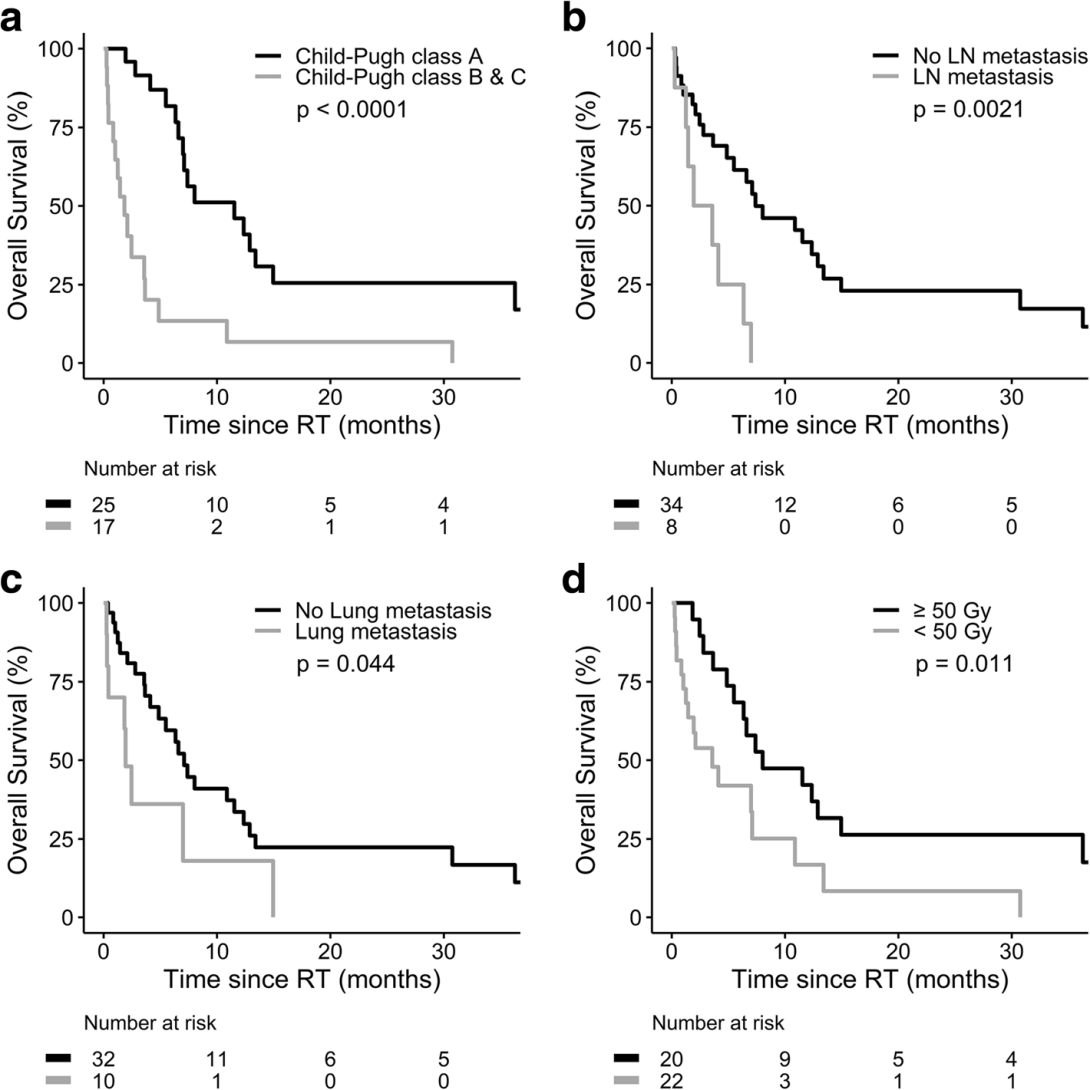
Overall survival by (**a**) Child-Pugh class, (**b**) lymph node (LN) metastasis, (**c**) lung metastasis before radiotherapy, and (**d**) radiotherapy dose (EQD_10/2,_ equivalent dose in 2-Gy fractions, α/β = 10)

**Fig. 3:**
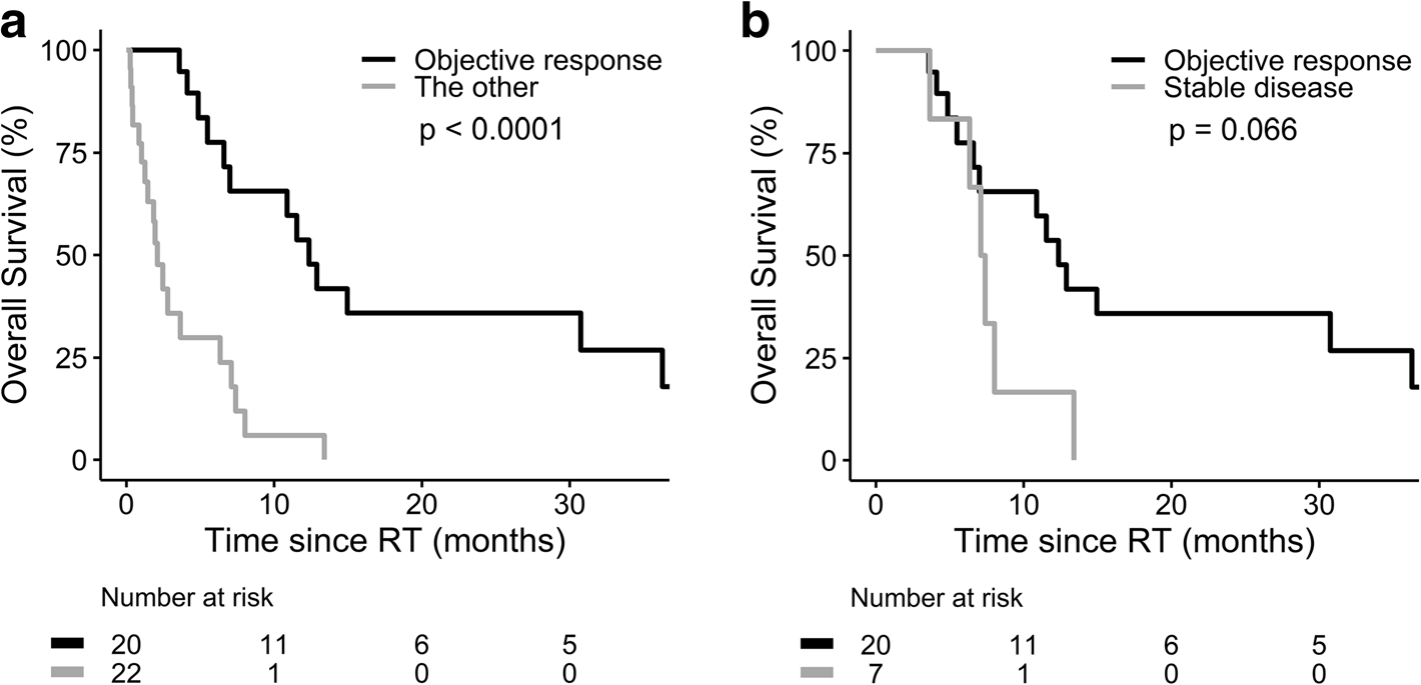
Overall survival by IVC response in (**a**) whole 42 patients, and (**b**) 27 evaluable patients

The median follow-up time was 4.4 months (range, 0.26–55.89) in the whole cohort. Fifteen patients died before CT or MRI for the response evaluation. Among the 27 evaluable patients, CR, PR and SD were achieved in four, 16 and seven patients, respectively. For the entire cohort, the objective response rate was 47.6%. The 1-year OS rate was 30.0%, with a median survival of 6.6 months (95% CI 3.7–9.5; Fig. 1). The OS was not associated with RT techniques (2.1 and 7.1 months in 3DCRT and IMRT group, respectively, *p* = 0.33). On the hand, the median OS was significantly longer for patients with Child-Pugh class (CPC) A (11.5 vs. 1.8 months, *p* < 0.0001; Fig. 2a), without LN metastasis (7.4 vs. 1.9 months, *p* = 0.002; Fig. 2b), without lung metastasis (7.1 vs. 1.9 months, *p* = 0.044; Fig. 2c), and RT EQD_10/2_ ≥ 50 Gy (8.0 vs. 3.6 months, *p* = 0.011; Fig. 2d). Furthermore, the objective response of IVC was associated with a longer median OS in the entire cohort (12.4 vs. 2.1 months, *p* < 0.0001; Fig. 3a). Among the 27 evaluable patients, there was a trend toward better OS in the objective responder of IVC (*p* = 0.066; Fig. 3b). Multivariate analysis confirmed that CPC, LN metastasis, lung metastasis and objective response of IVC thrombus were independent predictors for OS (Table 4).

**Table 4:** Univariate and Multivariate Analysis of Covariables Associated with Overall Survival

Variable	Univariate analysis		Multivariate analysis	
	HR (95% CI)	*P* value	HR (95% CI)	*P* value
Age (< 65 vs. ≥65 years)	0.709 (0.350–1.434)	0.338		
Gender (female vs. male)	0.748 (0.502–1.114)	0.153		
ECOG performance status (2–3 vs. 0–1)	1.656 (1.138–2.410)	0.008	1.175 (0.365–3.776)	0.787
Child-Pugh class (B&C vs. A)	2.123 (1.472–3.062)	0.000	2.411 (1.073–5.414)	0.033
Right atrium invasion (yes vs. no)	1.330 (0.905–1.952)	0.146		
Portal vein invasion (yes vs. no)	1.961 (1.343–2.864)	0.000	1.086 (0.662–1.782)	0.743
Hepatic vein invasion (yes vs. no)	0.970 (0.662–1.422)	0.876		
LN metastasis (yes vs. no)	1.926 (1.232–3.010)	0.004	2.118 (1.171–3.831)	0.013
Lung metastasis (yes vs. no)	1.509 (1.000–2.276)	0.050	1.751 (1.064–2.883)	0.028
Systemic treatment^a^ (without vs. with)	2.430 (1.625–3.634)	0.000	1.346 (0.680–2.667)	0.394
RT dose (EQD_10/2_, Gy)^b^ (<50 vs. ≥50)	1.583 (1.100–2.280)	0.013	1.050 (0.669–1.649)	0.832
Objective response of IVC^c^ (no vs. yes)	2.326 (1.552–3.488)	0.000	2.604 (1.544–4.391)	0.000

Abbreviations: *ECOG* Eastern Cooperative Oncology Group, *LN* lymph node, *RT* radiotherapy, *IVC* inferior vena cava

^a^Systemic therapy given concurrently with and/or after RT.

^b^Equivalent dose in 2 Gy fractions, α/β = 10

^c^Objective response: complete and partial responses

### Lung metastasis and pulmonary embolism

**Fig. 4:**
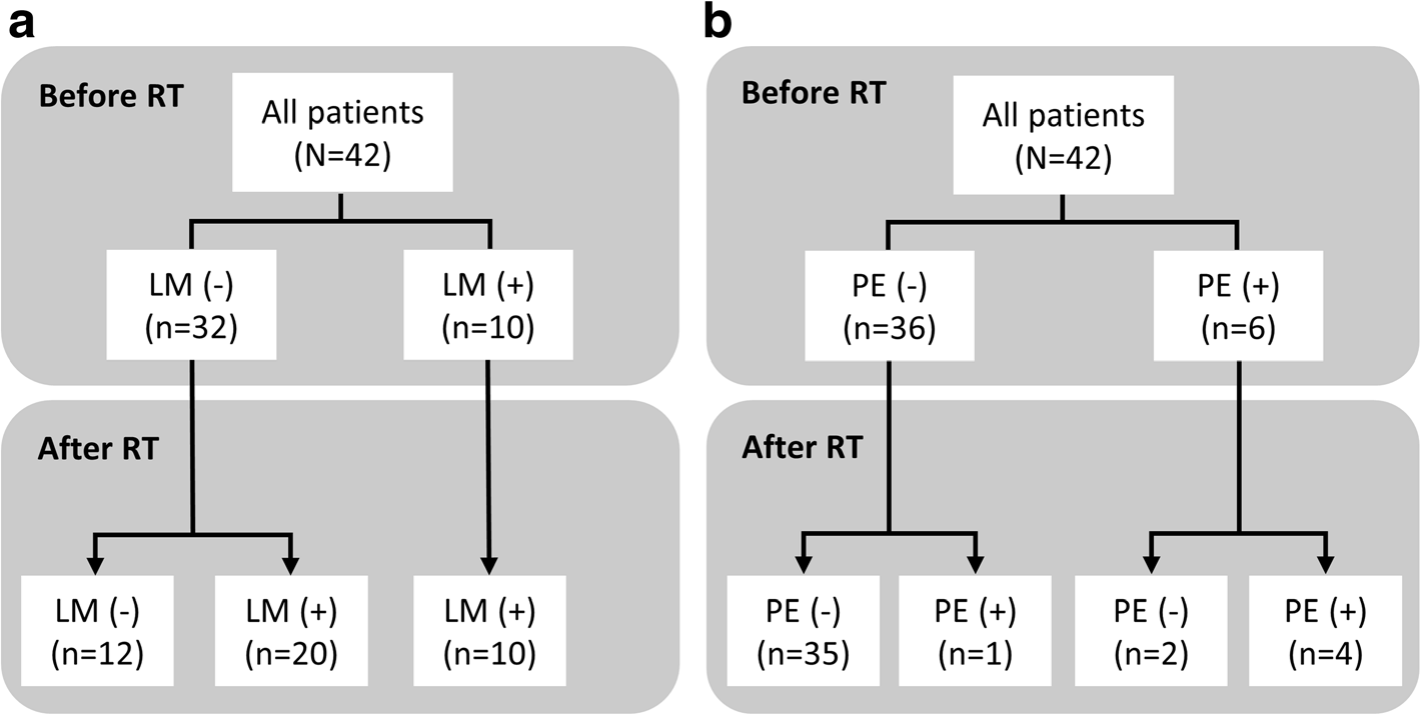
Status of (**a**) lung metastasis (LM), and (**b**) pulmonary embolism (PE) before and after RT

**Fig. 5:**
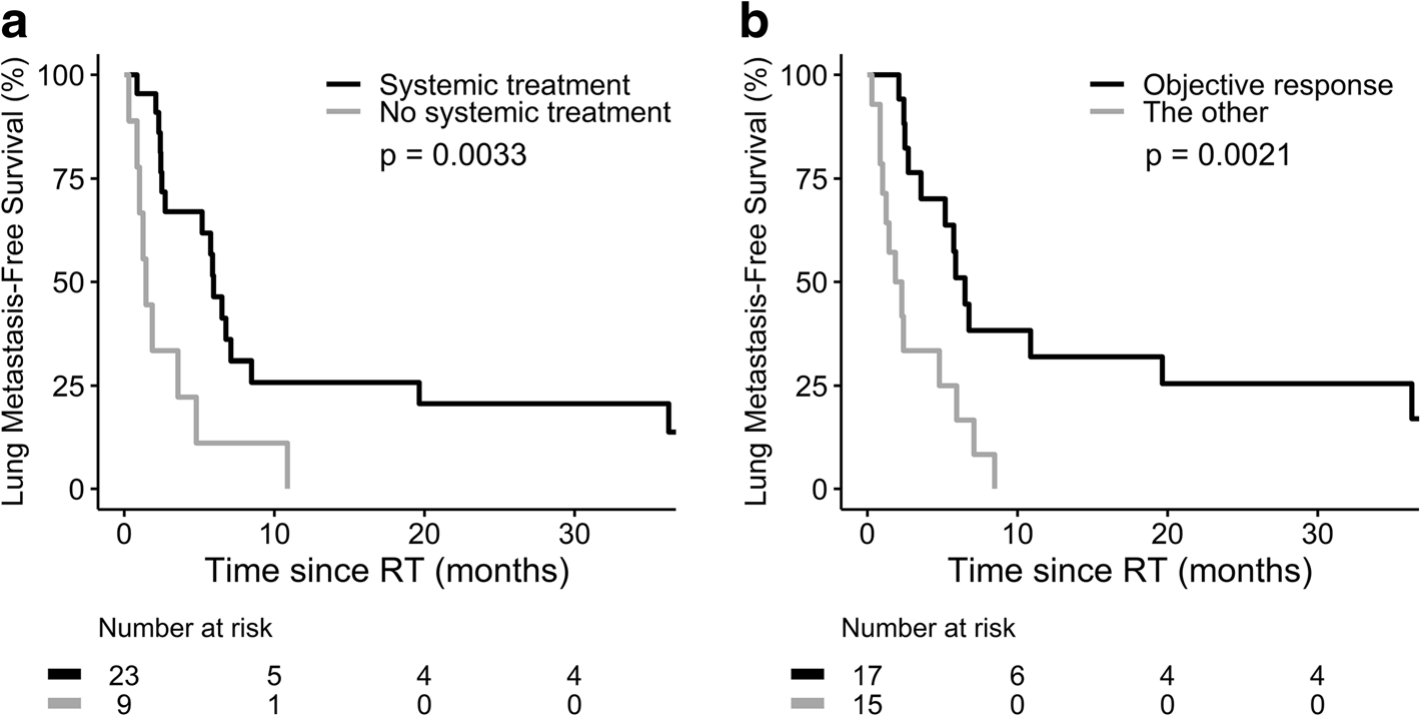
Lung metastasis-free survival by (**a**) use of systemic treatment concurrent with and/or after RT, and (**b**) IVC response in 32 patients without lung metastasis before RT

Ten (23.8%) patients had lung metastasis before RT to IVC thrombus. During post-RT follow-up, lung metastasis was identified in additional 20 patients (Fig. 4a). Among the 32 patients without lung metastasis before RT, use of systemic therapy concurrently with and/or after RT was associated with a longer median lung metastasis-free survival (5.9 vs. 2.1 months, *p* = 0.0033; Fig. 5a). By multivariate analysis, objective response of IVC thrombus was the only independent predictor for lung metastasis-free survival (Table 5). Patients with objective response of IVC thrombus had a longer median lung metastasis-free survival (6.5 vs. 1.9 months, *p* = 0.002; Fig. 5b). Furthermore, six (14.3%) patients had pulmonary embolism before RT. During post-RT follow-up, resolution of pulmonary embolism was noted in two patients who received anticoagulant and systemic treatment (1 with sorafenib followed by ramucirumab, and 1 everolimus followed by sorafenib). On the other hand, one of 36 patients without pulmonary embolism before RT had pulmonary embolism during post-RT follow-up (Fig. 4b).

**Table 5:** Univariate and Multivariate Analysis of Covariables Associated with Lung Metastasis-Free Survival

Variable	Univariate analysis		Multivariate analysis	
	HR (95% CI)	*P* value	HR (95% CI)	*P* value
Age (< 65 vs. ≥65 years)	0.745 (0.344–1.617)	0.457		
Gender (female vs. male)	0.664 (0.284–1.552)	0.344		
ECOG performance status (2–3 vs. 0–1)	4.069 (1.665–9.943)	0.002	3.337 (0.895–12.440)	0.073
Child-Pugh class (B&C vs. A)	3.336 (1.475–7.543)	0.004	2.847 (0.867–9.354)	0.085
Right atrium invasion (yes vs. no)	2.134 (0.906–5.028)	0.083		
Portal vein invasion (yes vs. no)	2.614 (1.109–6.162)	0.028	1.149 (0.406–3.250)	0.794
Hepatic vein invasion (yes vs. no)	0.973 (0.436–2.172)	0.947		
LN metastasis (yes vs. no)	4.360 (1.415–13.429)	0.010	1.016 (0.248–4.171)	0.982
Systemic treatment^a^ (without vs. with)	3.263 (1.332–7.988)	0.010	2.463 (0.919–6.600)	0.073
RT dose (EQD_10/2_, Gy)^b^ (<50 vs. ≥50)	2.171 (0.962–4.896)	0.062		
Objective response of IVC^c^ (no vs. yes)	3.471 (1.498–8.042)	0.004	5.732 (1.906–17.235)	0.002

Abbreviations: *ECOG* Eastern Cooperative Oncology Group, *LN* lymph node, *RT* radiotherapy, *IVC* inferior vena cava

^a^Systemic therapy given concurrently with and/or after RT.

^b^Equivalent dose in 2 Gy fractions, α/β = 10

^c^Objective response: complete and partial responses

### Patterns of failure

At the data cutoff, 33 (78.6%) patients died. The site of first progression was shown in Table 6. The most common site was lung in 14 (33.3%) patients. Only three (7.1%) patients had their first progression in the IVC thrombus. The most common causes of death were liver failure due to tumor progression or hepatic decompensation in 11 (33.3%) and lung metastasis in seven (21.2%) patients.

**Table 6:** Site of First Progression and Cause of Death

Disease	No. of patients (%)
Site of first progression	
IVC thrombus	3 (7.2)
Liver	9 (21.5)
Distant metastasis	21 (50.0)
Lung	14 (33.3)
Brain	3 (7.1)
Bone	2 (4.8)
Others	2 (4.8)
Cause of death	
Liver failure	11 (33.3)
Lung metastasis	7 (21.2)
Pulmonary embolism	2 (6.1)
Variceal bleeding	2 (6.1)
Duodenal ulcer bleeding	2 (6.1)
Pneumonia	2 (6.1)
SBP	2 (6.1)
Brain metastasis	1 (3.0)
Acute renal failure	1 (3.0)
CVA	1 (3.0)
Unknown	2 (6.1)

Abbreviations: *SBP* spontaneous bacterial peritonitis, *CVA* cerebrovascular accident

### Toxicity

The most common acute toxicities were fatigue in 13 (31%), dysphagia in four (9.5%), radiation dermatitis in three (7.1%) and cough in one (2.4%) patient. These toxicities were grade 1 or 2. In addition, elevation of Child-Pugh score ≥ 2 were observed in nine (21.4%) patients.

## Discussion

HCC involving IVC is a challenging clinical condition. The optimal treatment remains undetermined. Previous studies suggested the potential therapeutic role of RT in these patients [6, 9–16]. In the present study, 42 HCC patients were treated with RT to IVC thrombus (7 with 3-DCRT and 35 IMRT). RT was earlier stopped in six patients (3 due to terminal liver disease, 2 pulmonary embolism and 1 respiratory failure). The remaining 36 patients completed RT with median EQD_10/2_ of 50 Gy. For all 42 study patients, the median OS was 6.6 months and 1-year OS rate was 30.0%. This result was similar to the experience of two Asian institutions [10, 11] but compared unfavorably with the pooled data from prior eight studies (1-year OS rate of 53.6%) [6]. We next examined the possible factors contributing to this dismal outcome. A multivariable analysis of patient characteristics demonstrated that CPC B/C, LN metastasis, lung metastasis and the absence of objective response of IVC thrombus were associated with poorer OS.

LN metastasis and liver cirrhosis are key prognostic determinants of HCC patients. In our cohort focusing on HCC involving IVC, eight patients with LN metastasis received individualized systemic treatment for the involved LN. These patients had a significantly inferior OS of 1.9 months when compared to those without LN metastasis. It is interesting to note that LN metastasis was still an independent predictor of survival in so advanced stage HCC. This result was in line with prior studies [9, 13]. Moreover, CPC is widely used for the clinical assessment of liver cirrhosis and liver function. In our study, patients with CPC A had a longer median OS time than those with CPC B/C (11.5 vs 1.8 months). Similar findings were disclosed in prior reports which showed a longer median OS of 12.2 months in patients with CPC A than 6.1 months in CPC B [9], and confirmed CPC A was an independent predictor for better OS [12]. Collectively, the results of our and previous studies indicated that the residual liver function was an important clinical factor in these patients who commonly have concomitant intra−/extra-hepatic lesions needing further therapies.

Lung is a common site of extrahepatic dissemination in HCC. Of 42 patients in the current study, ten patients had lung metastasis before RT. The presence of lung metastasis before RT was correlated with poor OS. To our knowledge, this is the first study to show lung metastasis before RT was an independent predictor for OS in HCC patients receiving RT to IVC tumor thrombus. Furthermore, lung metastasis was found in additional 20 patients during post-RT follow-up. From the present retrospective analysis, it is difficult to attribute these newly developed lung metastases to thrombus dislodgment during RT or the natural history of cancer. On the other hand, we showed that objective response of IVC thrombus was an independent predictor for lung metastasis-free survival. Systemic therapy given concurrently with and/or after RT was associated with a longer lung metastasis-free survival in 32 patients who did not have lung metastasis before RT. This finding suggested the potential role of systemic therapy on reducing lung metastasis after RT to IVC tumor thrombus.

Advances in radiation technology made it possible to deliver higher RT dose to the tumor without causing severe complications. In the present study using modern RT for IVC tumor thrombus, patients receiving RT EQD_10/2_ higher than 50 Gy had a longer median OS time than those of lower dose. Moreover, objective response of IVC thrombus to RT was an independent predictor for better OS. This finding was consistent with the result of the prior study [13]. On the other hand, RT-related adverse effects were major concerns in these HCC patients with advanced stage and limited survival. Pulmonary embolism was suspected to be a complication due to thrombus dislodgment during RT in one study [11]. In the current cohort, pulmonary embolism was identified in six patients before RT. Among the remaining 36 patients, only one patient had pulmonary embolism during post-RT follow-up. This finding suggested pulmonary embolism was more likely a common natural consequence of IVC thrombus than complication of RT. However, we cannot exclude the possibility that this newly developed pulmonary embolism of our cohort was due to RT-induced thrombus dislodgment. Aside from this possible adverse event of pulmonary embolism, no other severe side effect was noted in the present study. This rarity of severe complications might be due to underestimation in our retrospective review for patients with short survival time. But on the positive side, the low complication rate may reflect the advantage of 3DCRT and IMRT which delivered relatively low RT dose to normal organs.

Our study has several limitations. First, the study is retrospective and carries with it all of the biases inherent in such an analysis. Specifically, treatment related morbidity might be underestimated due to insufficient information in the medical record. Second, the number of enrolled patients was small. It is possible to miss significant relationships from the data. Finally, our study investigated patients treated between 2007 and 2018. Changes in therapies for HCC and supportive management over time may affect patient outcome and contribute to study bias.

## Conclusions

In conclusion, this study showed RT was a feasible and safe treatment option for IVC tumor thrombus in HCC patients. Detailed information relating to pulmonary embolism and lung metastasis was provided in the present study. Pulmonary embolism due to thrombus dislodgment during RT rarely occurred. Use of systemic therapy given concurrently with and/or after RT was associated with a longer lung metastasis-free survival. Based on our results, RT might be a treatment option incorporated into combination therapy for HCC involving IVC.

## Abbreviations

3DCRT: Three dimensional conformal radiation therapy
CI: Confidence interval
CPC: Child-Pugh class
CR: Complete response
CT: Computed tomography
CTV: Clinical target volume
ECOG: Eastern Cooperative Oncology Group
EQD_10/2_: Equivalent dose in 2-Gy fractions, α/β = 10
GTV: Gross tumor volume
Gy: Gray
HCC: Hepatocellular carcinoma
IMRT: Intensity-modulated radiotherapy
IVC: Inferior vena cava
LN: Lymph node
MRI: Magnetic resonance imaging
OS: Overall survival
PD: Progressive disease
PE: Pulmonary embolism
PEI: Percutaneous ethanol injection
PR: Partial response
PTV: Planning target volume
RFA: Radiofrequency ablation
RT: Radiotherapy
SD: Stable disease
TACE: Transarterial chemoembolization
TAE: Transarterial embolization
